# Femoral Aneurism Cured by Compression

**Published:** 1851-06

**Authors:** 


*Femoral Aneurism cured by Compression.*—Mr. Smyly communicated to the Surgical Society of Ireland, November 23, 1850, the following case of femoral aneurism, successfully treated by compression:—

"Patrick O'Gorman, aged 48, a schoolmtster, came under my care in April, 1847, three years and a half ago. He stated that, in August, 1846, eight months before I saw him, when walking at his ordinary gait, he suddenly lost, in a great measure, the use of his right leg, and with great difficulty got on half a mile further. Next day he could not put his foot under him, or leave his bed. He suffered intense pain in the middle of the thigh, in the knee, and down the outside of his leg. It was so severe as to prevent him leaving his bed for a week. It then abated, but has returned occasionally, with more or less severity ever since. He is a married man, sober, and well conducted. He was admitted into the Meath Hospital on the 14th of April, 1847. A pulsating tumour, the size of an egg, having all the characteristics of aneurism, existed in the lower part of the middle third of the right thigh. His general health was good. There was excitement of the circulation, and a slight bruit de soufflet at the heart. In consultation with my colleague, it was determined to treat this case by compression, so applied as not to interrupt completely the flow of blood in the femoral artery. The patient being bled, and confinement to the recumbent position being strictly enjoined, pressure was made at two different points alternately, by means of two clamps—one applied to the external iliac artery, the other to the femoral. This treatment was persevered in with more or less assiduity for nearly three months, with no other advantage than a diminution in the size of the tumor. Weary of the restraint, he desired to leave the hospital; and as he fully understood the use of the instruments, and the plan to be pursued, he was permitted to go out. He was then appointed schoolmaster to the National School at Blackrock, and continued to fulfil his arduous duties for about three months, when, having walked a distance of two miles, he suddenly felt a severe pain in the tumor, extending down the leg. The pain continued unabated for two days, and then gradually subsided. Seven days after the occurrence of the severe pain, he called upon me. On examining the tumor, all pulsation had ceased. This he found to have taken place two days ago, the contents of the tumor to have become consolidated, and all pain to have disappeared. Three months after the cessation of pulsation, I had an opportunity of examining the patient: the tumor, though diminished in size, was still to be felt about as large as a walnut, and very hard.

"November 15, 1850, I called upon him, and found him in the enjoyment of good health, equal to perform the duties attendant upon the management of a large school. A very small hard tumor is still traceable at the seat of the aneurism, and pain is sometimes felt down the outside of the leg. On examining the heart and large arteries, no morbid sound is audible; they are apparently free from disease."

*Reflections.*—1st. When we consider the protracted period required to accomplish a cure in this case, we learn not to abandon hopes of a cure when our efforts are baffled, and prove unavailing, even for six months.

2d. We find the excitement of the circulation and the bruit de soufflet to subside and disappear on the cure of the aneurism just as similar sympathetic affections are relieved when the cure is affected by ligature. This case, then, meets the objection urged against the treatment by compression—viz., that disease of the heart is more likely to attend it than when a ligature is applied; for we see the same beneficial result follow the cure of the aneurism in this as in those cases cured by ligature.—*Dublin Med. Press, December* 11, 1850, from *Am. Jour. Med. Sci.*

